# A novel visual marker to distinguish haploids from doubled haploids in rice (*Oryza sativa, L*) at early growth stages

**DOI:** 10.1186/s13007-023-01085-z

**Published:** 2023-12-01

**Authors:** Chaitanya Ghalagi, Malavalli Rajashekar Namratha, Kavita Kotyal, Shiva Prakash, Basavaiah Mohan Raju

**Affiliations:** Department of Crop Physiology, University of Agricultural Sciences, GKVK, Bangalore, 560065 India

**Keywords:** Acute leaf apex, Androgenic rice, Attenuate leaf apex, Doubled haploids, Haploids, Visual markers

## Abstract

Doubled haploid technology, which enables the generation of homozygous lines in a single step, is one of the modern tools being employed for accelerating breeding processes in different crops. In rice, a globally important staple food crop, doubled haploid production through androgenesis is increasingly being employed in breeding programs. Amongst the androgenic rice lines, doubled haploids are formed spontaneously at about 50–60%, while the remaining 40–50% of plants remain as haploids. As haploids cannot be easily identified, it is routine to grow all the rice androgenic lines till maturity and harvest the seeds from the fertile doubled haploids. Therefore, the methods that facilitate easy identification of haploids at an early developmental stage in rice would enable treatment of such haploid lines with colchicine, to increase the efficiency of doubled haploid production. Further, it would also help in eliminating the operational cost involved in maintaining them till maturity. In the above context, a systematic study to identify easily observable physiological and morphological differences between haploid and doubled haploid rice lines was undertaken. Rice haploids were found to be noticeably different from doubled haploids in photosynthetic rate, transpiration rate, stomatal conductance, and morphology of lodicules, stigma and style, features which have not been reported before. Most importantly, rice haploids invariably have acute leaf apex which is easily distinguishable from the doubled haploids that have attenuated leaf apex shape. Very high per cent accuracy in the prediction of ploidy level was observed when haploids were identified at an early developmental stage based on leaf apex shape, and the results verified with flow cytometry perfectly matches with leaf apex shape. The study establishes ‘acute leaf apex’ shape as an accurate visual marker to rapidly identify haploid rice lines at an early developmental stage in a cost-effective manner.

**Supplementary Information:**

The online version contains supplementary material available at 10.1186/s13007-023-01085-z.

## Background

Plant breeding is the art and science of making genotypes with new combinations of alleles and involves two broad steps. First, crossing of the parents with the desired alleles and second, the ‘fixation’ of the alleles so that the new combination is carried faithfully to the subsequent generations. While the first process takes only one generation, the second process typically involves 6–8 cycles of repeated selfing, which takes at least  2–4 years if not more of breeding time. However, using doubled haploid technology, one can ‘fix’ the new allelic combination in just one generation, shortening the breeding cycle by 2–4 years. In addition, doubled haploid technology is known to increase genetic gain [33]. Therefore, doubled haploid technology is commonly employed to accelerate the breeding programs of different commercial crops for which the technology is available, both in public institutes and commercial companies.

Rice is one of the important crop plants where doubled haploidy is extensively used for breeding new varieties. In this crop, in vitro androgenesis is efficient and hence, is the commonly used method for doubled haploid production [22]. During in vitro androgenesis, haploid cells often undergo “spontaneous haploid genome duplication” to give rise to doubled haploids [31]. This phenomenon is an inherent tendency observed in many plant species including rice. The frequency of this spontaneous chromosome doubling in rice varies with genotype hovering around an average of 50–60% [25], and the rest, 40–50% of the androgenic regenerants remain haploids. Thus, the plants derived from androgenesis are a mix of haploids, Doubled haploids and mixoploids [15]. As the haploids cannot be distinguished easily from doubled haploids, all the androgenic regenerants in rice are grown till maturity and seeds are harvested from the fertile doubled haploids and the sterile haploids are discarded [12]. 

Convenient tools for the identification of haploids at early growth stages of androgenesis would be of great value. First, the operational expenses involved in growing till the maturity of the haploids, which almost constitute 50% of plants produced, could be saved. Second, the identified early-stage haploids can potentially be subjected to artificial chromosome doubling treatments to recover additionally doubled haploids and, thus, increase the overall doubling efficiency.

Several methods of determining the ploidy of androgenic plants are available. They can broadly be classified as direct (chromosome counting) or indirect (flow cytometry, stomatal size, chloroplast number in the guard cells, pollen morphology or plant morphology) methods. Chromosome counting is the direct and precise method for analyzing the ploidy level of androgenic regenerants, which could be done in cells undergoing either mitotic or meiotic cell division. Even though chromosome counting is the most reliable method for ploidy determination [2], this method is time-consuming, requires a well-equipped laboratory, a qualified work team, and is technically challenging and error-prone [40, 41], which limits its use in large scale commercial production [2].

Flow cytometry, on the other hand, is an accurate, rapid and simple option for a large-scale ploidy determination in early phases [29]. It also allows the detection of mixoploid (plants with unequal number of chromosomes in adjacent cells or tissues) regenerants. However, the cost involved in the preparation of high-quality plant DNA samples and the need of expensive equipment are the hurdles that account for its low utilization in plant breeding (Table 3, [11]). Other approaches for rapid differentiation of haploids from doubled haploids are, counting the number of stomata per unit leaf area, measuring the length and width of stomatal cells, and determining the density of chloroplasts per stomatal guard cell [28]. However, these methods too are time-consuming, require a well-equipped laboratory, a qualified work team, and are error-prone to employ in rice. Yet another method to identify haploids is to assess pollen fertility/sterility in regenerants through staining of pollen grains with aceto-carmine or tetrazolium, or fluorescein diacetate [10, 19]. Pollen grains of haploid plants show aberrant morphology of little staining with reduced or no protoplasm, while the pollen from diploids displays intact cell walls with deeply stained protoplasm [16]. However, pollen grains are produced only in the mature plants and hence, this method cannot be used to distinguish haploids at early stages of production. Lastly, haploid plants grow slowly and are relatively short, with a smaller number of tillers. The length and width of leaves, as well as panicles, are smaller in haploids when compared to doubled haploid plants [37]. However, such morphological evaluations, though cost-effective, rapid and easy to perform, cannot be employed in the early stages of plant growth. Further, its efficiency is dependent on environmental conditions and on stability of morphological attributes throughout the plant growth and development [1].

In contrast to rice, many phenotypic markers to distinguish haploids from doubled haploids have been developed in maize (corn). *E.g.*, the anthocyanin color marker, R1-nj (Navajo) is one of the most widely used markers in a variety of haploid inducer systems [4, 27]. Differences in the oil content between haploid and diploid kernels have also been developed as a haploid marker in corn [26, 35]. Lastly, triple anthocyanin color markers expressed in the seedling roots and leaf sheaths [3] are being widely used in corn. These early phenotypic markers in corn have greatly helped in subjecting the haploids to chromosome-doubling treatments and producing a large number of doubled haploids for employing in breeding programs in corn. However, such phenotypic markers for identifying haploid plants are lacking in rice.

In the present study, we undertook a systematic investigation to identify characters that can differentiate haploids from doubled haploids at the early stages of rice androgenesis. Several distinct and previously not-reported features of haploid rice plants, like the leaf apex shape, photosynthetic rate (*A*), transpiration rate (*E*), and ligule and pistil morphology were identified. Further, the investigation demonstrated the utility of the ‘shape of leaf apex’ as a visible marker to accurately and quickly differentiate haploids from doubled haploids at early stages of rice doubled haploid production in a cost-effective way (Additional file 7: Table S2).

## Results

**Fig. 1:**
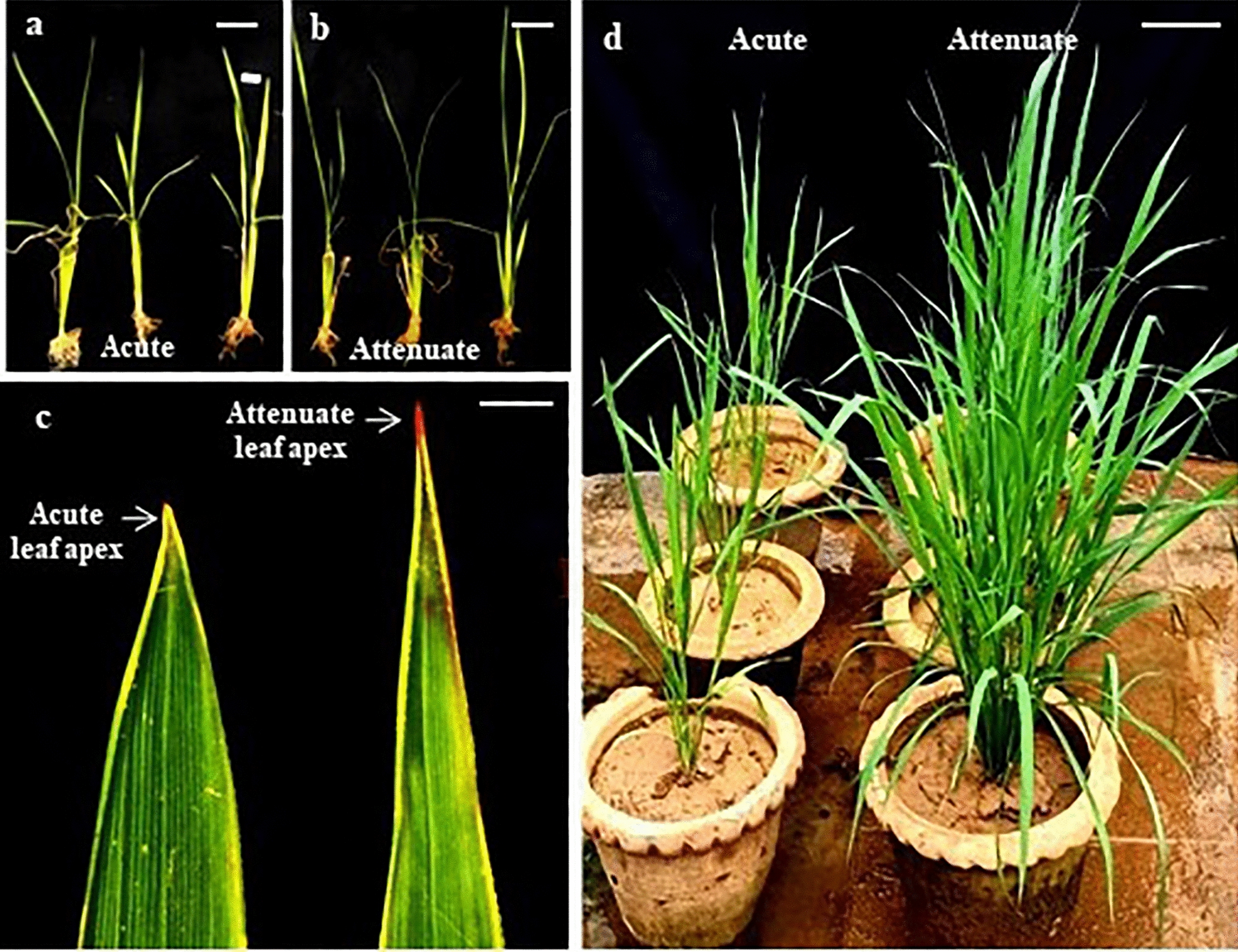
Leaf apex shape of androgenically derived rice lines at post hardening and late vegetative stages. **a, b** Representative samples of androgenic plants at post hardening stage grouped based on leaf apex shape, Scale = 4 cm. **c** Acute and Attenuate leaf apices, Scale = 0.3 cm.  **d** Phenotypes of the same lines shown in Fig. 1 a and 1 b, at late vegetative stage, Scale = 6 cm

In rice anther culture, a mix of haploids and doubled haploids are produced which appear more or less similar in the early developmental stages (Fig. 1a, b). In the present study, we studied different morphological (leaf apex shape, plant height, tiller number, floral parts) and physiological characters (photosynthetic rate, stomatal conductance, transpiration rate and chlorophyll content) of all the regenerants at various developmental stages to develop a convenient method to identify haploids from doubled haploids and compared it with seed set and widely used flow cytometry method.

### Two distinct leaf apices were observed amongst androgenic regenerants of rice

In the first set of plants, 52 out of 63 androgenic regenerants derived from KRH-4 rice hybrid grew taller, produced fertile spikelets and set seeds to indicate their doubled haploid nature, while the remaining 11 plants were short, bushy and produced sterile spikelets which did not set seeds and hence, were concluded to be haploids. These 63 plants were carefully examined to identify any morphological differences which were previously not reported. Among all the characters studied, two distinct leaf apex shapes, viz., acute and attenuate (Fig. 1c), were observed amongst the 63 regenerants. Interestingly, all the 52 lines that set seeds had attenuate leaf apex, while the 11 sterile plants had invariably acute leaf apex.

As the leaf apex shape can be easily distinguished at very early stages of growth, the second set of 137 androgenic plants upon careful examination found to have 67 plants with attenuate leaf apex while, the remaining 70 had acute leaf apex shape at post-hardening stage. After approximately 2-months of growth, all the 67 lines belonging to the attenuate leaf apex group set seeds while, remaining 70 plants marked as belonging to acute leaf apex were sterile and did not set seeds   (Table 1). A representative sample of leaf apices documented in the study is shown in Additional file 1: Fig. S1 and Additional file 2: Fig. S2. Similar observations were also made in 300 androgenic lines derived from a cross between TIL-14 and AC39000 where, 55 lines with attenuate leaf apex set seeds while, the remaining 245 plants with acute leaf apex did not set any seeds (Table 1). These results confirmed that, leaves of haploid rice plants exhibit a distinct acute shaped apex. To confirm whether normal diploids show any variability in leaf apex shape, observations were made on 200 IRRI germplasm accessions. All the 200 diploid germplasm lines invariably had attenuate leaf apices (Table 1, Additional file 2: Fig. S2), similar to those leaves borne on doubled haploid lines.

**Table 1:** Leaf apex type and seed set in androgenic rice lines and in diploid germplasm collection

Sl. No	Material	Leaf Apex	No. of lines observed	Leaf apex shape (%)	No. of lines showing sterility	No. of plants showing seed set
1	Androgenic lines from KRH-4 (Commercial hybrid)	Attenuate	119	59.50	0	119
		Acute	81	40.50	81	0
2	Androgenic lines from TIL-14 X AC39000	Attenuate	55	18.33	0	55
		Acute	245	81.66	245	0
3	IRRI Germplasm Accessions	Attenuate	200	100.00	0	200
		Acute	0	0.00	0	0

χ^2^ = 325.63**, χ^2^ (0.01, 2df) = 9.210 **Significant at 1% level

Three lines out of the 300 androgenic lines derived from a cross between TIL-14 and AC39000 lines found to have with both ‘attenuate leaf apex’ tillers and tillers with ‘acute leaf apex’ exhibiting their mixaploid nature (Fig. 5). Interestingly, within these lines, the tillers which exhibited attenuate leaf apex shape set seeds and the remaining acute leaf apex tillers did not set seeds at maturity (Fig. 5).

### Morphological differences of androgenic lines differing in leaf apex shape during vegetative growth

**Fig. 2:**
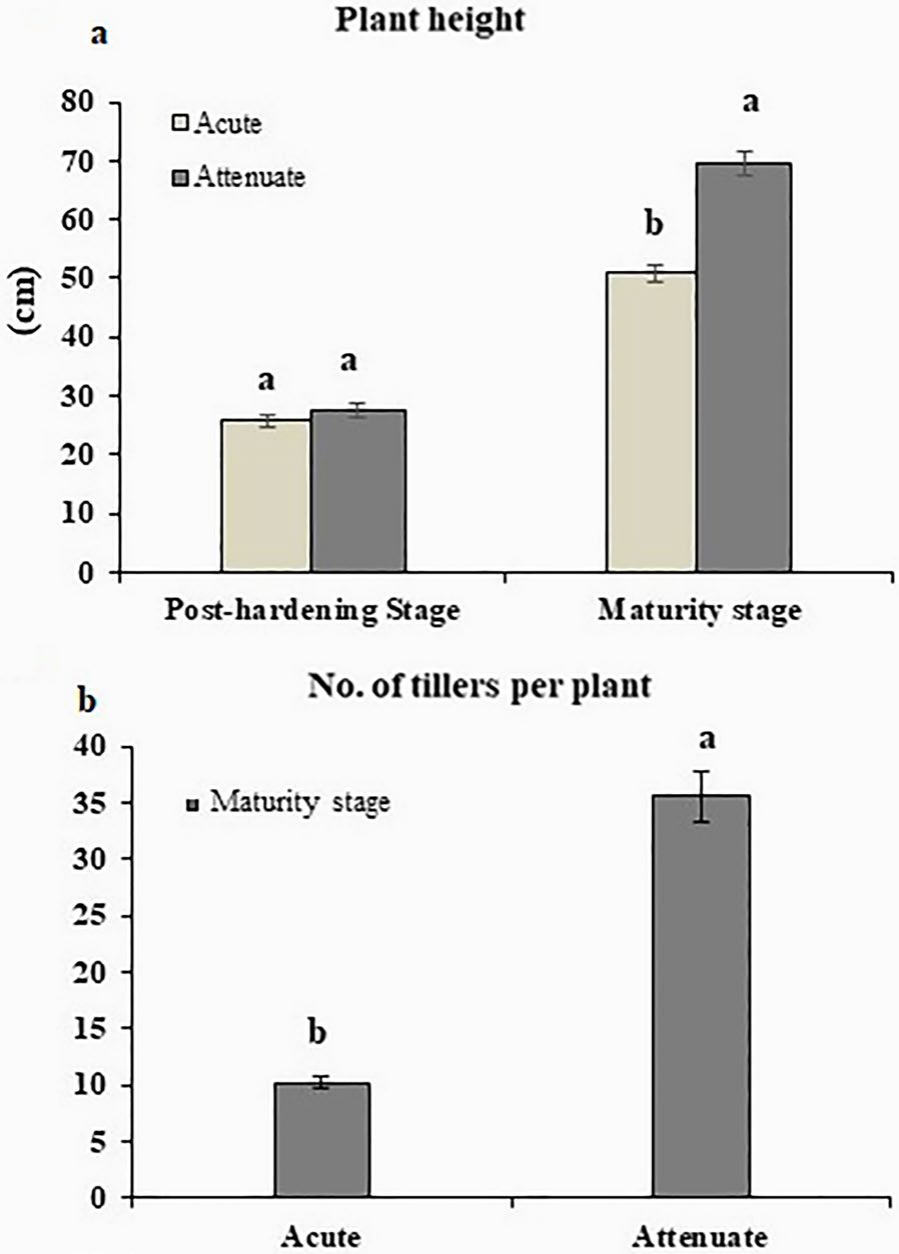
**a** Plant height at post-hardening and maturity stage, **b** Tiller number at maturity stage of rice androgenic lines differing in leaf apex shape (n = 10, p < 0.05)

At post-hardening stage, regenerants with acute leaf apex did not appear to be different with respect to plant height when compared to regenerants with attenuate leaf apex (Figs. 1a, b, 2a). However, when these regenerants were grown to maturity, the plants with acute leaf apex were noticeably dwarf (A characteristic feature of being smaller in size compared to normal diploid plants of the same species) with few tillers which are typical of haploid phenotypes (Figs. 1d, 2b).

At post-hardening stage, mean leaf area was higher in androgenic plants with acute leaf apex (4.43 cm^2^) as compared to lines with attenuate leaf tip (3.10 cm^2^), which was due to significantly higher leaf width in acute (0.46 cm) than attenuate (0.34 cm) leaves (Table 2). However, the leaf length did not differ significantly between the two groups of plants at this developmental stage. Leaf index (the ratio of leaf length to leaf width) was higher in attenuate leaf apex (38.80) as compared to acute leaf apex (26.77) (Table 2).

**Table 2:** Leaf morphology parameters of androgenic plants differing in the leaf apex

Androgenic plants	Leaf area (cm^2^)	Leaf length (cm)	Leaf width (cm)	Leaf index	Leaf apex area (cm^2^)
Post-hardening stage					
Attenuate leaf apex	3.10 ± 0.47^b^	12.21 ± 1.66^a^	0.34 ± 0.03^b^	38.8 ± 2.51^a^	0.380 ± 0.01^b^
Acute leaf apex	4.43 ± 0.35^a^	11.94 ± 1.36^a^	0.46 ± 0.04^a^	26.77 ± 0.84^b^	0.55 ± 0.02^a^
Maturity stage					
Attenuate leaf apex	37.22 ± 3.92^a^	32.55 ± 2.31^a^	1.50 ± 0.07^a^	21.85 ± 1.45^a^	0.324 ± 0.01^b^
Acute leaf apex	15.74 ± 2.18^b^	21.06 ± 1.64^b^	0.96 ± 0.07^b^	22.33 ± 1.26^a^	0.620 ± 0.04^a^

Student t-test was used to compare the means of two groups of plants (attenuated and acute leaf apex)

Mean ± standard error; a and b letters indicate a significant difference in Duncan’s multiple range test at p < 0.05

Means are averages of 10 replicates of androgenic regenerant lines

Leaf apex area refers to the area of the leaf measured in the region 1 cm from the tip

**Fig. 3:**
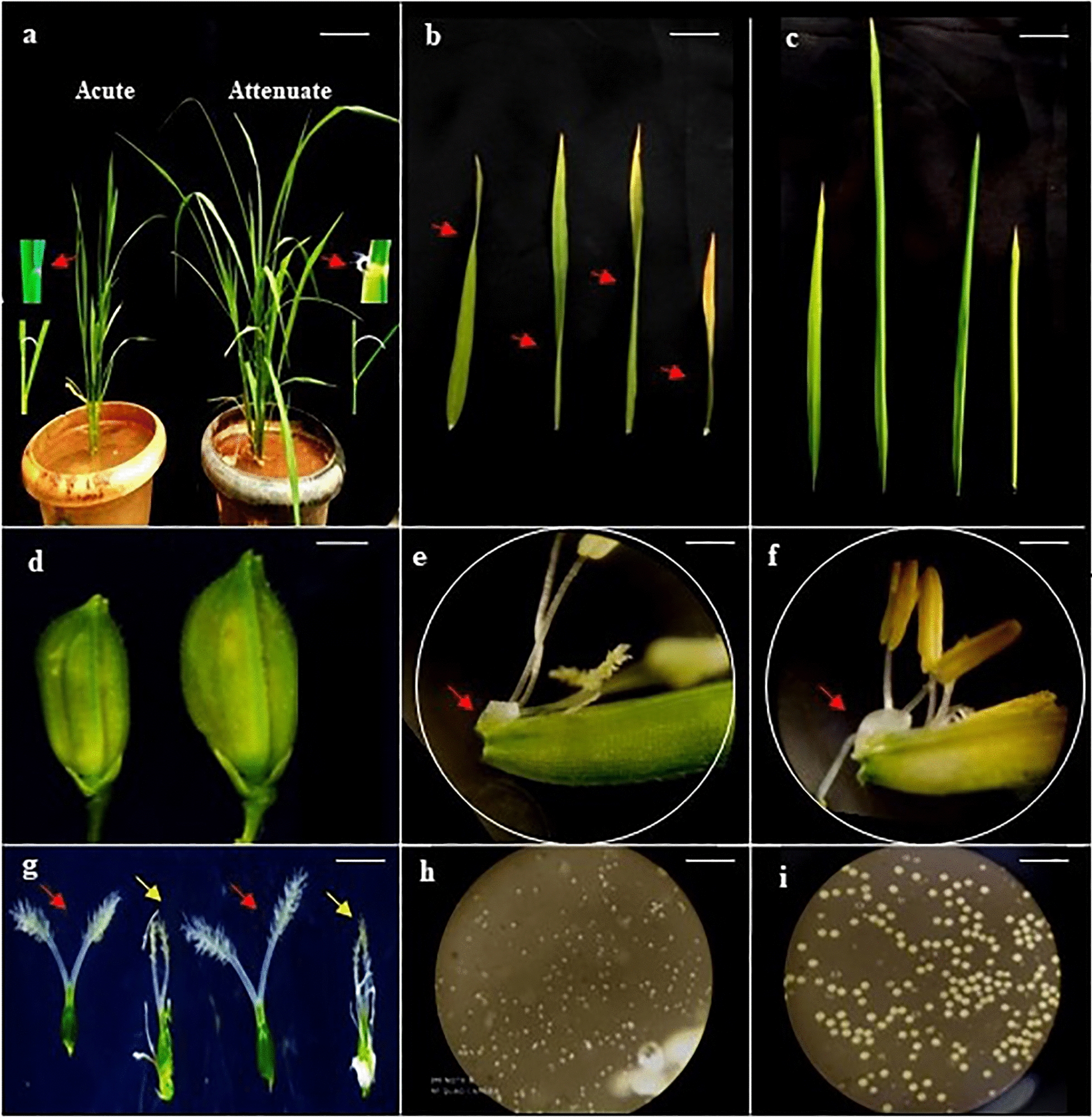
Morphological differences between androgenic lines differing in leaf apex shape at late vegetative stage and reproductive stage. **a** Erect leaf of acute leaf apex (left) vs. droopy leaves of attenuate leaf apex (right) of androgenic plants at early vegetative stage. Scale = 6 cm. The auricles and ligules observed in the lines are shown in the respective insets. **b** twisted nature of leaf blades of lines with acute leaf apex,. Scale = 1.7 cm. **c** non-twisted nature of leaf blades in lines with attenuate leaf apex, Scale = 1.7 cm. **d** Spikelet on acute (left) and attenuate leaf apex (right) lines, Scale = 1.5 mm. **e** Deformed lodicule of acute leaf apex line, Scale = 0.75 mm. **f** Normal swollen lodicule of attenuate leaf apex line, Scale = 0.75 mm. **g** Pistils of lines with acute leaf apex (yellow arrow) and attenuate leaf apex (red arrow) lines, Scale = 0.5 mm. **h** Pollen grains in acute leaf apex lines, Scale = 0.39 μm. **I** Pollen grains in attenuate leaf apex lines, Scale = 0.39 μm

At reproductive stage, leaf area, leaf length and leaf width were lower in acute apex leaves (15.74 cm^2^, 21.06 cm and 0.96 cm) when compared to attenuate apex leaves (37.22 cm^2^, 32.55 cm and 1.50 cm). The area of the terminal 1 cm leaf apex of attenuate leaf apex was lower than acute leaf apex at hardening (0.380±0.01 cm^2^ as against 0.550±0.02 cm^2^) as well as at maturity stage of plant growth (0.324±0.01 cm^2^ as against 0.620±0.04 cm^2^). Further, most of the haploid leaves were erect (Fig. 3a) with twisted leaf blade (Fig. 3b) which was noticeable even in early vegetative stages of growth, a phenomenon not observed in leaves of doubled haploid lines. In addition, auricles were rudimentary and ligules were reduced in lines with acute leaf apices as compared to auricles and ligules observed in lines with attenuate leaf apex at later stages only (Fig. 3a inset).

### Physiological differences of androgenic plants differing in leaf apex shape

**Fig. 4:**
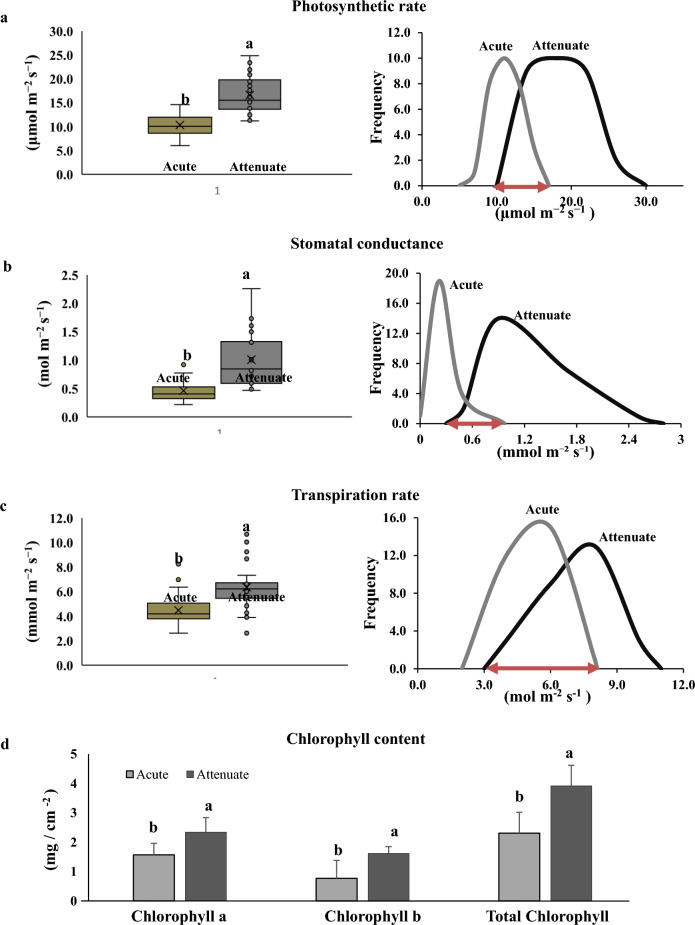
Physiological differences between androgenic lines (Acute and Attenuate) differing in leaf apex shape at early vegetative stage. **a** Photosynthetic rate, **b** Stomatal conductance, **c** Transpiration rate and **d** Chlorophyll (a, b and total) content (n = 30, p < 0.05). Note: Overlapping of Acute and Attenuate plants for photosynthetic rate (*A*) range between 10 to 17 μmol m^−2^ s^−1^, stomatal conductance (g_*s*_) between 0.3 to 0.97 mol m^−2^ s^−1^ and transpiration rate (*E*) between 3 to 8.1 mmol m^−2^ s^−1^

Attempts to measure the physiological differences in androgenic plants differing in ‘leaf apex shapes’ at post-hardening stage was not successful owing to the small size of leaves. Hence, photosynthetic responses and chlorophyll pigment composition were measured at mid-vegetative stage. Mean photosynthetic rate of doubled haploids was 1.6 times that of haploids (Additional file 6: Table S1). However, there was considerable variation within the androgenic haploid or doubled haploid lines and the rate of photosynthesis (*A*) in many doubled haploids were less than that of a few haploid lines. Specifically, 8 haploids and 7 doubled haploids fell in the overlapping region (10 to 17 μmol m^−2^ s^−1^) in the frequency distribution graph of photosynthetic rate (Fig. 4a and Additional file 6: Table S1). Transpiration rate (*E*) followed a similar trend as photosynthetic rate. That is, though the mean transpiration rate of doubled haploids was 1.4 times that of haploids, a large proportion of haploid and doubled haploid lines overlapped in their individual transpiration rates (Fig. 4c and Additional file 6: Table S1). Mean stomatal conductance (g_s_) of doubled haploids was 2.19 times that of haploids. To a large extent, haploids had a low and a distinct g_s_ (Fig. 4b). Still, of the total 62 androgenic lines observed, 8 haploid lines and 7 doubled haploid lines had overlapping stomatal conductance readings (Fig. 4b and Additional file 6: Table S1).

Chlorophyll a, b and total chlorophyll content were significantly higher in attenuated tip leaves (2.34 mg g^−1^, 1.62 mg g^−1^ and 3.92 mg g^−1^) than in acute tip leaves (1.57 mg g^−1^, 0.77 mg g^−1^ and 2.31 mg g^−1^) (Fig. 4d).

### Morphological differences in floral parts of rice androgenic lines differing in leaf apex shape

At reproductive stage, androgenic plants with acute leaf apex showed abnormalities in floral organs typical of haploid plants. Spikelet size of these plants was drastically reduced when compared to spikelet size of lines with attenuated leaf apex (Fig. 3d). Anthers produced on lines with acute leaf apex were pale yellow or white and reduced in size (Fig. 3f). Ovary and style were deformed (Fig. 3g) and pollen grains were sterile (Fig. 3h) in all acute leaf apex plants which never was observed in lines with attenuate leaf apex. At anthesis, pollen dehiscence was clearly visible in all the attenuate leaf apex lines which was not observed in any of the acute leaf apex lines. However, in all the acute leaf apex lines, the lodicules were deformed (Fig. 3e) which resulted in non-opening of spikelets in acute leaf apex plants.

### Flow cytometric analysis of rice androgenic lines differing in leaf apex shape

A set of fifty randomly chosen androgenic rice lines at the post-hardening stage, when subjected to flow cytometric analysis, showed almost a perfect correlation between ‘acute leaf apex’ and haploidy as well as ‘attenuate leaf apex’ and doubled haploidy with an exception of line no. K-459 which exhibited the leaf apex morphology of acute shape. Although K-458 and K-459 have similar ploidy as shown by flow cytometry data, our scoring of the leaf apex morphology differed between these two lines (Additional file 3: Fig. S3b). However, the overall correlation between the leaf apex prediction and flow cytometry results shows approximately 98% at an early growth stage viz., the post-hardening stage (Additional file 3: Fig. S3a and S3b). Upon reaching maturity, all the plants identified as doubled haploids through morphological observations as well as flow cytometry analysis, successfully set seeds except for K-267 and K-307 (these two lines had only chaffy seeds). Conversely, none of the lines identified as haploids through flow cytometry were able to set seeds (Additional file 3: Fig. S3a and S3b).

## Discussion

Doubled haploid breeding is one of the efficient means to improve crop germplasm [17, 36, 42]. It enables rapid stacking and screening of recombinant haplotypes in fixed genetic backgrounds [7] bypassing the six generations of single-seed decent that is typically required to produce near-inbred lines. It has various economic and logistic advantages over conventional inbred lines, in addition to genetic benefits [5]. For successful adoption of this technology in any breeding program, it is necessary to develop a large number of doubled haploid lines. In rice, androgenic way of doubled haploid generation is widely followed. The plants derived from androgenesis are a mix of diploids, haploids, doubled haploids and mixaploids [15]. The frequency of spontaneous chromosome doubling in rice has been reported to range between 50 and 60% [25]. Our results showed a spontaneous doubling efficiency ranging between 20 and 30% while nearly 70–80% of androgenic plants remained as haploids. Thus, there is a very wide scope for increasing the doubling efficiency of rice, if haploids are identified early and subjected to diploidization (Additional file 5: Fig. S5). Currently, it is routine to grow all androgenic rice plants till maturity, harvest the seeds from the fertile doubled haploids and discard the sterile haploid plants which incidentally constitute more than 50% of the plants produced. Therefore, convenient methods of identification of haploids at an early stage during rice androgenesis, like immediately after hardening of regenerants, facilitates their diploidization to increase the doubled haploid production and also minimizes the operational cost and effort involved in maintaining haploid plants till maturity.

**Fig. 5:**
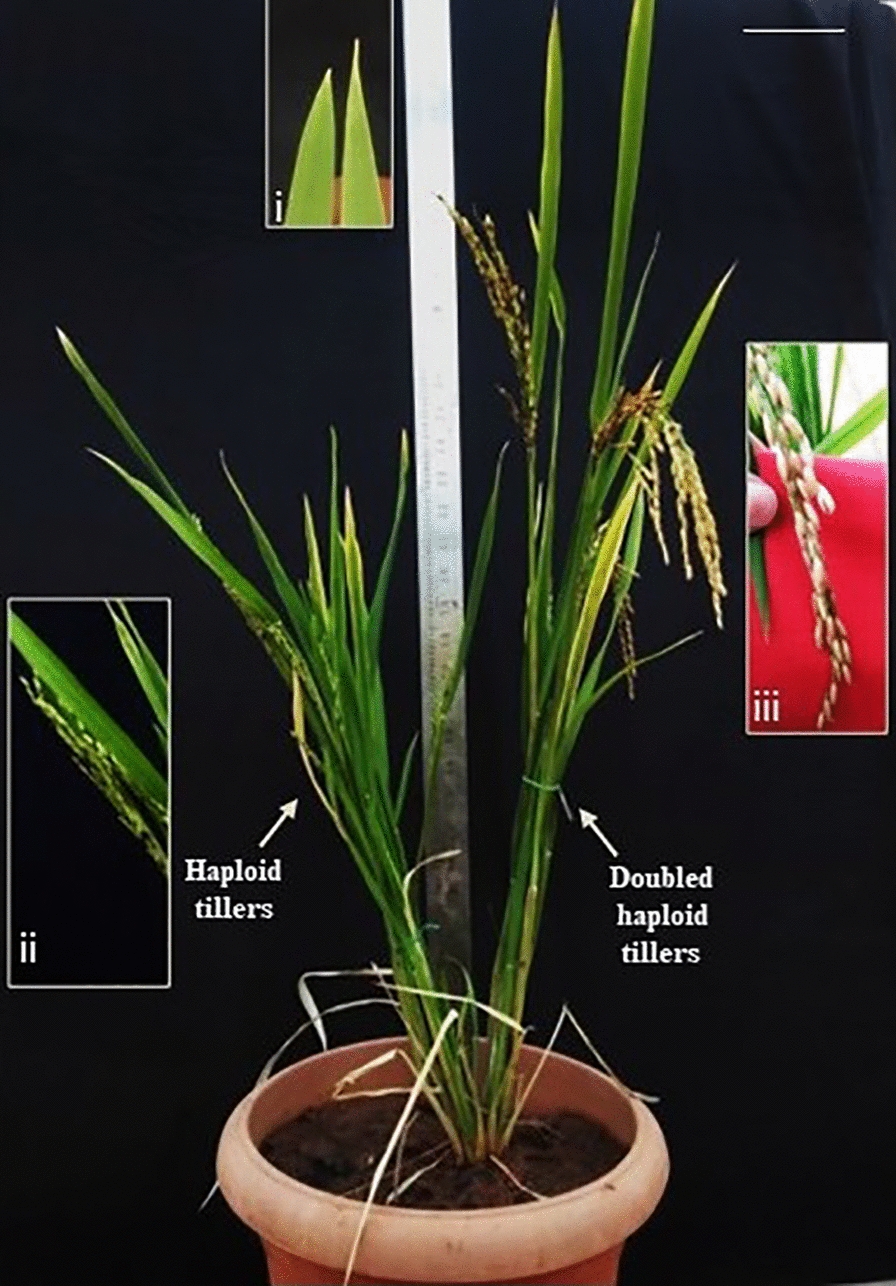
Chimeric plant having tillers with acute leaf apex and tillers with attenuate leaf apex. Scale = 6 cm. Inset: (i) Acute and attenuate leaf apex, (ii) Acute leaf apex tillers did not set seeds and (iii) Tillers with attenuate leaf apex set seeds

### Leaf apex shape as a visual marker for haploid lines identification at early stages in rice

In the present investigation, flow cytometry analysis of fifty randomly chosen androgenic lines established almost perfect correlation between leaf apex shape and ploidy (Additional file 3: Fig. S3a and Fig. S3b). That is, all the lines with attenuate leaf apex were doubled haploids, while those with acute leaf apex were haploids. Further, all lines with acute leaf apex, identified and grown separately from early developmental stage exhibited known phenotypic characters of rice haploids in later developmental stages viz., erect leaves, extensive tillering with short and bushy appearance (Fig. 1). Further, within the chimeric androgenic lines (mixaploids), the tiller with attenuate leaf apex produced only fertile seeds while, the spikelets of acute leaf apex tiller of the same line were sterile (Fig. 5). In addition, there was no variability of leaf apex shape in the 200 rice germplasm lines that were observed (Additional file 2: Fig. S2) and all of them invariably had attenuate leaf apex, like the doubled haploids/diploid lines. Taken together, the above evidences demonstrate that the leaf apex shape can be effectively employed to identify haploids from doubled haploids at a very early growth stage.

Seed set is another confirmatory feature to differentiate doubled haploids from haploids [12, 23]. In the present study, the acute leaf apex rice plants were of short stature and produced only sterile spikelets. Interestingly, line no. K-459 showed the ploidy of doubled haploids through flow cytometry analysis although the leaf apex morphology was of acute leaf apex shape. However, the extent of correlation between the leaf apex morphology and ploidy assessed through flow cytometry remains to be very high (98%) suggesting that the leaf apex morphology can be a unique marker to identify the haploids at earlier stages. The attenuate leaf apex plants grew tall and produced fertile spikelets which in turn set seeds. Thus, there was a tight correlation between the acute and attenuate leaf apex plants with the known phenotypic features of haploids and doubled haploids respectively (Fig. 1d, Additional file 1: Fig. S1, Additional file 3: Fig. S3a and Fig. S3b and Table 1). However, amongst the attenuated leaf apex plants, two of the lines (K-267 and K-307) did not set seeds but turned out to be doubled haploids in flow cytometry results (Additional file 3: Fig. S3a and Fig. S3b). Thus, some of the doubled haploid lines too may not set seeds due to tissue culture effect or other environmental factors. This indicates that ‘seed set criteria’ is not a full proof feature of doubled haploids. Moreover, ‘seed set criteria’ cannot be employed to differentiate haploids from doubled haploids till the plant matures and hence, cannot be employed for identification of haploids intended for chromosome doubling. Thus, in rice, leaf apex shape marker is 98%  accurate as flow cytometry and practically more useful than ‘seed set’ to distinguish haploids amongst androgenic rice plants.

In contrast to rice, maize haploids and doubled haploids did not show differences in leaf apex shape (data not shown). Though this needs to be verified in other grass species, it can be concluded that distinct leaf apex shape of haploids is not a universal phenomenon.

The cause of two distinct leaf apex shapes in androgenically derived rice lines, which occur roughly in equal ratio, is likely because of differences in ploidy of the regenerants and not due to segregation of alleles of any genes. This is evidenced by our results where all the acute leaf apex lines were haploids, while the parental lines and all the doubled haploid lines showed attenuate leaf apex. Also, a germplasm survey of rice did not reveal any variability of leaf apex shape with all the accessions bearing leaf with attenuate apex. Therefore, the underlying cause of haploids having a distinct leaf apex needs to be investigated at cellular level from the perspective of leaf morphogenesis.

### Photosynthetic rate / transpiration rate / stomatal conductance for differentiating haploids from doubled haploids

Several studies in multiple plant species, have demonstrated significantly lower stomatal chloroplast number in haploid plants compared to doubled haploids, and has been recommended as a tool for haploid / diploid distinction [21, 32]. Stomatal guard cell size also has been associated with the ploidy of cells [8]. However, counting chloroplasts or measuring stomatal length under microscope is labor intensive and error prone. We examined whether the reduced chloroplast number and stomatal size in haploid lines results in lower photosynthetic rate or decreased transpiration rate, either of which can conveniently be measured with a handheld portable photosynthesis system (IRGA). To the best of our knowledge, such comparative studies of haploids and doubled haploids with respect to physiological parameters have not been reported in literature.

In general, the photosynthetic rate (Fig. 4a) or transpiration rate (Fig. 4c) and stomatal conductance (Fig. 4b) of haploids were much reduced as compared to the doubled haploids. As all the androgenic rice lines were grown in the same ambient conditions, the lower rate of photosynthesis observed in haploids (Fig. 4) could be because of lower chloroplast number / chlorophyll content (Fig. 4d). In addition, the lower transpiration rate observed in the haploids (Fig. 4c), points towards reduced gas exchange also contributing to the reduced photosynthesis rate, which in turn might be a result of smaller sized and less densely distributed stomata in haploids as compared to doubled haploids or diploid plants (Additional file 4: Fig. S4).

Though the current investigation demonstrated that there was a significant difference between the haploids and doubled haploids in the mean photosynthetic rate, mean transpiration rate and mean stomatal conductance (Fig. 4a–c), there are practical difficulties in employing these parameters to distinguish haploids from doubled haploids. For example, it is known that environmental conditions that the plants have been exposed to before photosynthetic rate measurements, impact data leading to difficulty in data interpretation [24]. It is also reported that, any differences in growth conditions, plant age and measurement method can bring about different results of photosynthetic rate [44]. In addition, photosynthetic rate or transpiration rate is dependent on the time of the day. Most importantly, the haploid lines and doubled haploid lines fall into a range in their physiological characteristics and a number of individual haploid lines in the upper region of the range overlap with the doubled haploids lines falling in the lower end of their range (Fig. 4a–c). Among the three physiological parameters investigated, though *g*_*s*_ was the best marker, it was not completely error free in unequivocally identifying haploids (Fig. 4b). In contrast, leaf apex character of rice plants, being qualitative, falls into two distinct non-overlapping groups – either acute or attenuate. Thus, the speed of identifying the leaf apex shape and the possibility of scoring without any instrument makes the leaf apex based identification of haploids superior as compared to the physiological parameter based methods.

Though there are numerous studies showing genetic variations in photosynthetic traits in both crop and wild species [14], variability in photosynthetic rate, stomatal conductance and transpiration rate amongst doubled haploids has not been the subject of any previous investigations. The wide variability in the photosynthetic rate (11–24 μmol m − ^2^ s^−1^) or stomatal conductance (0.5–1.78 mol m^−2^ s^−1^) or transpiration rate (2.62–11 mmol m^−2^ s^−1^) observed amongst the androgenic doubled haploid lines reported in this study is interesting. In comparison, previously, a range of 12.8–25.5 μmol m^−2^ s^−1^ in photosynthetic rate has been observed in a population of introgression lines from a cross between upland rice and lowland rice [18] while, Ouyang et al. [30] had reported a range of 0.15–0.31 mol m^−2^ s^−1^ in stomatal conductance within a group of 6 rice cultivars (two cultivars each from upland rice, lowland rice and aerobic rice). Therefore, exploiting natural genetic variation in photosynthesis has been proposed to facilitate the development of cultivars with greater yield potential [13]. Further studies are needed to explore the utility of the variability of physiological parameters created in doubled haploids for exploitation in breeding programmes.

### Other markers to differentiate haploids from doubled haploids

The distinct morphology of lodicules and stigma in the haploids observed in the present study have not been reported previously (Fig. 3). Phenotypic characters specific to haploid individuals, like their smaller stature as compared to diploids/doubled haploids, partly because of their smaller cell size has been previously reported [10]. Haploid plants are also reported to generally produce smaller flowers with varying degrees of premature bud abscission, abnormal bud formation, irregular and uneven anther development, degenerated stigma and anther, or decreased pollen viability and fertility [16, 19, 38, 41]. However, all the above markers of haploids are either difficult to observe or occur only at flowering stage when the plants cannot be effectively subjected to colchicine treatment.

There have been previous efforts in rice to induce chromosome doubling in haploid lines at early growth stages using colchicine [43]. Increased doubling efficiency up to 29.4% and 35.1% has been reported by Wong [43] and Hooghvorst et al. [20], respectively. However, such attempts relied on flow cytometry for identifying the haploids, which is expensive and hence, have not been deployed in commercial rice doubled haploid programs. Our demonstration of leaf apex morphology as an accurate, simple, cost-effective phenotypic marker for identifying haploids, amongst rice androgenic lines at an early developmental stage (Additional file 7: Table S2), would help in subjecting them to colchicine treatment and thus, increase frequency of doubled haploid production. At a fundamental level, we are studying whether the observed reduced transpiration rate in haploid rice lines confers moisture stress tolerance as compared to diploid rice. In addition, we are investigating whether the large variability that was observed in the photosynthetic rate and transpiration rate can be employed in breeding program. Furthermore, the molecular and cellular basis of differences in leaf apex shape between haploids and doubled haploid rice lines remain to be explored.

## Conclusion and Perspective

In rice, doubled haploid technology involving in vitro anther culture has emerged as an efficient method of inbred line development. The development of androgenic plants, followed by identification of diploids, doubled haploids and haploids, and acclimatization and characterization of doubled haploids are the three important sequential stages in rice doubled haploidy (Additional file 5**:** Fig. S5). Although the development of more androgenic plants employing a standardized in vitro protocol is necessary to maximize doubled haploid efficiency, it is also important to recognize doubled haploid and haploid plants at various stages of development. About 50–60% of androgenic lines in rice are known to show spontaneous chromosome doubling while the rest remains as haploids which are currently identified at maturity and discarded. As such, indica rice cultivars are known to be recalcitrant and yield only a few androgenic lines, and the easy and inexpensive method of identifying haploids amongst androgenic lines at an early developmental stage would enable subjecting them to treatment with chromosome doubling chemicals and thus, increase doubled haploid production (Additional file 5**:** Fig. S5). The process would simultaneously help in avoiding the cost involved in maintaining the haploids till maturity. In this direction, the present study identifies the leaf apex shape in androgenic rice lines as a unique visual marker to distinguish haploids from doubled haploids with near hundred percent accuracy at an early developmental stage. The doubled haploid lines invariably bear leaves with attenuate leaf apex while the haploid lines have acute leaf apex. Despite the fact that the molecular mechanism of this variation in leaf apex between haploids and doubled haploids is unknown and necessitate a thorough elucidation, the detection of haploids using a visual marker (the shape of the leaf apex) is rapid and does not require expensive equipment like flow cytometer and therefore, forms as a high throughput approach (Additional file 7: Table S2).

## Material and methods

### Plant material

Haploids and doubled haploids of rice were produced by employing previously reported anther culture protocols with slight modifications [9]. Factors influencing the anther culture efficiency like panicle growth stage, microspore developmental stage, cold treatment, media composition and hormonal treatment were optimized for efficient callus induction and plant regeneration for the genotypes employed in the study [6]. A total of 500 androgenic regenerants, 200 of which derived from a commercial hybrid, KRH-4 and 300 lines derived from a cross between the breeding lines TIL-14 X AC39000, were employed in the study. The study also included 3 chimeric plants (which exhibited haploid and doubled haploid tillers on the same plant) and 200 (diploid) lines from IRRI germplasm collection being grown in the University of Agricultural Sciences, Bangalore campus.

Observations were recorded at different growth stages of rice. As there are no developmental stages defined for in vitro grown plants, we classified the stages for the convenience as shown below:

**Table:** 

Post-hardening stage	Post 10–15 days of hardening (3–5 leaf stage)
Early-vegetative stage	25–30 days after transfer to pots
Mid-vegetative stage	55–60 days after transfer to pots
Late-vegetative stage	75–80 days after transfer to pots
Reproductive stage	90–110 days after transfer to pots
Maturity	Plants with panicles having at least 85% of grains showing maturity

### Genetic detection of Diploids and their elimination

The plants produced through androgenesis have a mixture of haploids, spontaneously doubled haploids (SDH) and diploids. To separate out diploids from haploids and SDH, polymorphic SSR markers are generally used. Accordingly, in the present study polymorphic SSR markers were employed to identify diploids and eliminate them from the group of haploids and doubled haploids. To identify polymorphism, a total of 150 SSR markers were employed of which, 8 were found to be polymorphic for KRH-4 and 4 for TIL-14 X AC39000. The homozygous (haploids and doubled haploids) and heterozygous (diploids) plants were identified from among the anther regenerated plantlets using these 8 polymorphic markers. Initially, the banding pattern of parents was monitored and based on the banding pattern, polymorphic markers were identified. While the female and male parents give single but distinct bands, the F1 hybrid gives two bands each corresponding to one of the parents. Accordingly, when anther-derived plantlets were examined, the plantlets with bands similar to either of the donor parents were considered to be true haploids or spontaneous doubled haploids (SDH), and those with bands similar to parent hybrid were considered as diploids. Diploids were discarded and the remaining androgenic plants were subjected for ploidy analysis through leaf apex morphology and flow cytometry.

### Phenotypic characterization of androgenic plants

In the initial studies, ploidy of androgenic plants was assessed based on seed set at maturity (diploid plants derived from anther wall were identified through SSR markers and discarded at the beginning). Lines which produced seeds were classified as doubled haploids, while the sterile lines were classified as haploids. Such classified haploid rice plants were found to exhibit a specific shape of the leaf apex as compared to doubled haploid plants (Fig. 1). While in diploids/doubled haploid plants, leaf apex was observed to taper gradually to form a narrow-elongated tip, in haploids, the two edges of leaf remain more or less parallel till almost the tip of the leaf and ends in a sharp but not prolonged point (Fig. 1c). Based on the descriptions provided by NYBG Streere Herbarium on shapes of leaf apices [34], the observed haploid and doubled haploid leaf apices of rice were classified either as acute or as attenuate (Fig. 1).

The acute and attenuate leaf tip was distinguishable at post-hardening stage of androgenic rice plants. Hence, androgenic lines with attenuate leaf tip and those with acute leaf tips were segregated at post-hardening stage and used for morphological characterization at different developmental stages. Morphological parameters viz., plant height (cm) and leaf area (cm^2^) were recorded at post-hardening as well at maturity stage. The plant height was recorded from the base of the plant to the tip of the longest leaf using a meter scale. The leaf area was measured non-destructively, calculated as the product of the length and breadth of each leaf with which a correction factor of 0.75 was multiplied [45]. Number of tillers per plant was manually counted at maturity stage.

Area of the 1 cm leaf tip, was measured at post hardening and maturity stages, using a digital scanner (Epson perfection V 700 photo) with Win-RHIZO programme V. 2009 c 32-bit software.

Erect or droopy nature of leaves of androgenic plants was visually scored based on the degree of bending between leaf blade and leaf sheath (Fig. 3). Twisted or normal nature of leaf of androgenic plants was also visually scored (Fig. 3).

Ligules and auricle characters were recorded at reproductive stage. Morphological studies of floral parts of androgenic plants differing in leaf apex shape were carried out by dissecting the floral parts and observing under a Zeiss stereo-microscope.

### Photosynthetic rate, Transpiration rate and Stomatal conductance

Gas exchange parameters like Photosynthetic rate (*A*), Transpiration rate (*E*) and Stomatal conductance (*g*_s_) were measured with a portable gas-exchange system (LI-6400; LI-COR, Lincoln, NE) on the terminal, fully opened leaf at mid vegetative stage. All the measurements were recorded between 09 to 11 h under bright sunlight on pairs of an acute leaf apex line and an attenuate leaf apex line. The ambient CO_2_ concentration during the measurement ranged between 390–400 ppm, irradiance between 1200 and 1300 μmol m^−2^ s^−1^ (PAR) and average leaf temperature around 27–29 °C. On each of these pairs, observations were made on three different days, each day in a different order of the above said pairs, and the data of the three days were averaged.

### Chlorophyll content

Chlorophyll content was measured following the method of Shoaf and Lixm [39]. Briefly, 100 mg of fresh leaf tissue was chopped into small pieces, transferred to a glass tube containing10 ml dimethyl sulfoxide (DMSO). After incubating 24 h at room temperature, the samples were transferred to a boiling water bath and incubated for five minutes. Optical density of the supernatant was measured at 663 and 645 nm in UV–VIS Spectrophotometer. Chlorophyll-*a*, chlorophyll-*b* and total chlorophyll contents were estimated as previously reported [39].

### Ploidy analysis of androgenic plants using flow cytometry

Young leaves (1 inch) of randomly chosen attenuate and acute leaf apex lines, at post-hardening stage, were chopped with a sharp razor blade for isolation of nuclei in 1 ml extraction buffer (pH = 7.0) containing 25 μg/ml Propidium Iodide (PI), 0.1% Trisodium Citrate, 0.03% IGEPAL and 50 μg/ml RNase. The chopped leaf suspension was filtered through nylon filters with 30 μm mesh and incubated for 10–15 min before analysis in flow cytometer (BD FORTESSA X-20).

### Statistical analysis

The data generated in several experiments were analyzed statistically using SPSS programme. The normality of data was tested using Shapiro-wilk test and student’s t was used for estimating the significance of difference between the means of compared groups at 5% level of significance (P < 0.05).

### Supplementary Information

**Additional file 1: Fig. S1.** Representative samples of leaf apex shape of androgenically derived lines and their corresponding seed set. Red arrow points to the sharp tapering end of attenuate leaf.

**Additional file 2: Fig. S2.** Representative samples of leaf apex shape of germplasm accessions and their corresponding seed set.

**Additional file 3: Fig. S3a.** Representative samples of leaf apex shape of androgenically developed lines and their corresponding flow cytometry results. **b** Representative samples of leaf apex shape of androgenically developed lines and their corresponding ploidy level as determined by flow cytometry.

**Additional file 4: Fig. S4.** Variations in physiological parameters **a** Photosynthetic rate **b** Stomatal conductance **c** Transpiration rate, on three consecutive days among the androgenically developed lines differing in leaf apex shape at early vegetative stage.

**Additional file 5:** **Fig. S5.** Illustration of three important stages in rice doubled haploidy and significance of present study in increasing the efficiency of rice doubled haploids**.**

**Additional file 6: Table S1.** Gas exchange parameters of androgenically developed lines differing in leaf apex shape at early vegetative stage.

**Additional file 7: Table S2.** Comparison of visual (leaf apex) marker with two commonly used methods (flow cytometry and chromosome or plastid count) for identification of haploids in rice.

## Data Availability

The data supporting the findings of this study are available from the corresponding author.
