# Supplementary figures and images for: A novel visual marker to distinguish haploids from doubled haploids in rice (Oryza sativa, L) at early growth stages
Source: Plant Methods. 2023 Dec 1;19:137. doi: 10.1186/s13007-023-01085-z (PMC10691067; doi:10.1186/s13007-023-01085-z)

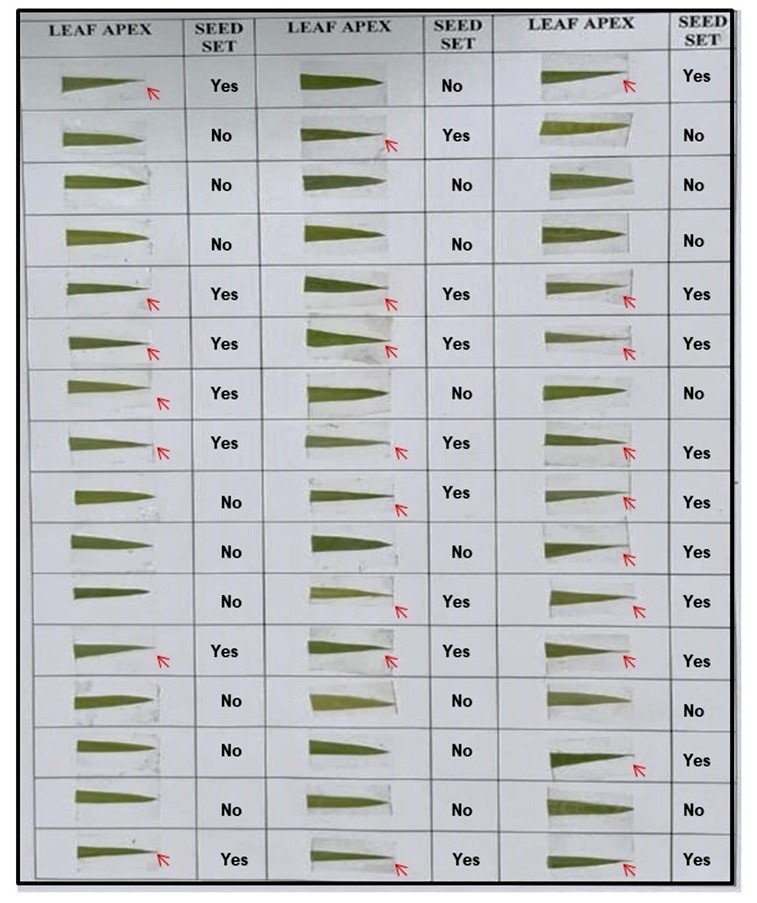

Supplement: Supplementary file 1 — Additional file 1: Fig. S1. Representative samples of leaf apex shape of androgenically derived lines and their corresponding seed set. Red arrow points to the sharp tapering end of attenuate leaf. [file 13007_2023_1085_MOESM1_ESM.jpg]

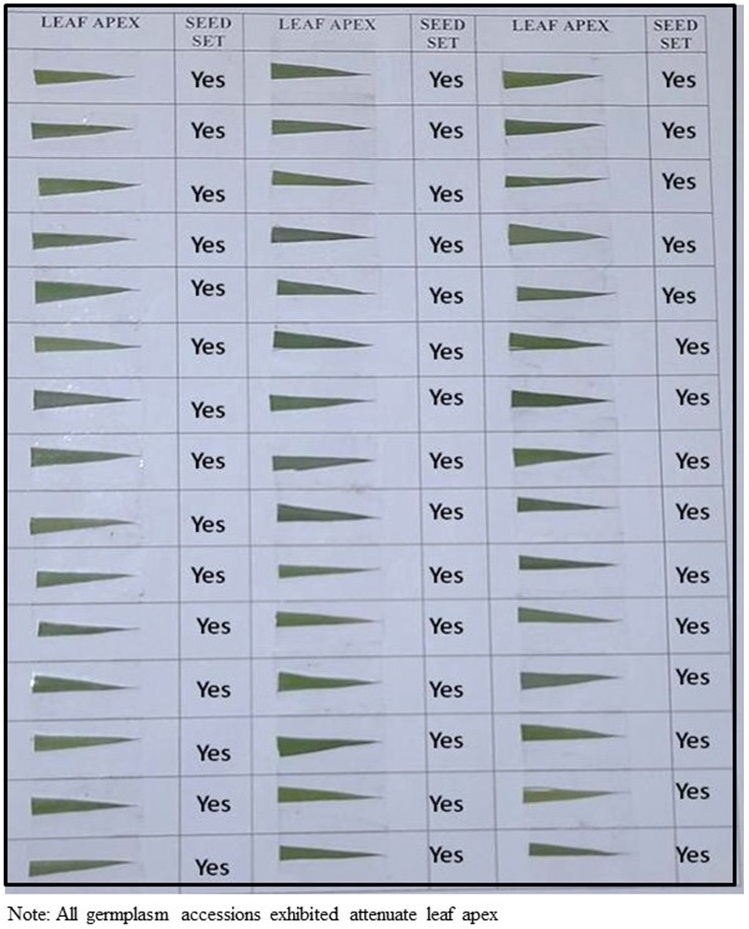

Supplement: Supplementary file 2 — Additional file 2: Fig. S2. Representative samples of leaf apex shape of germplasm accessions and their corresponding seed set. [file 13007_2023_1085_MOESM2_ESM.jpg]

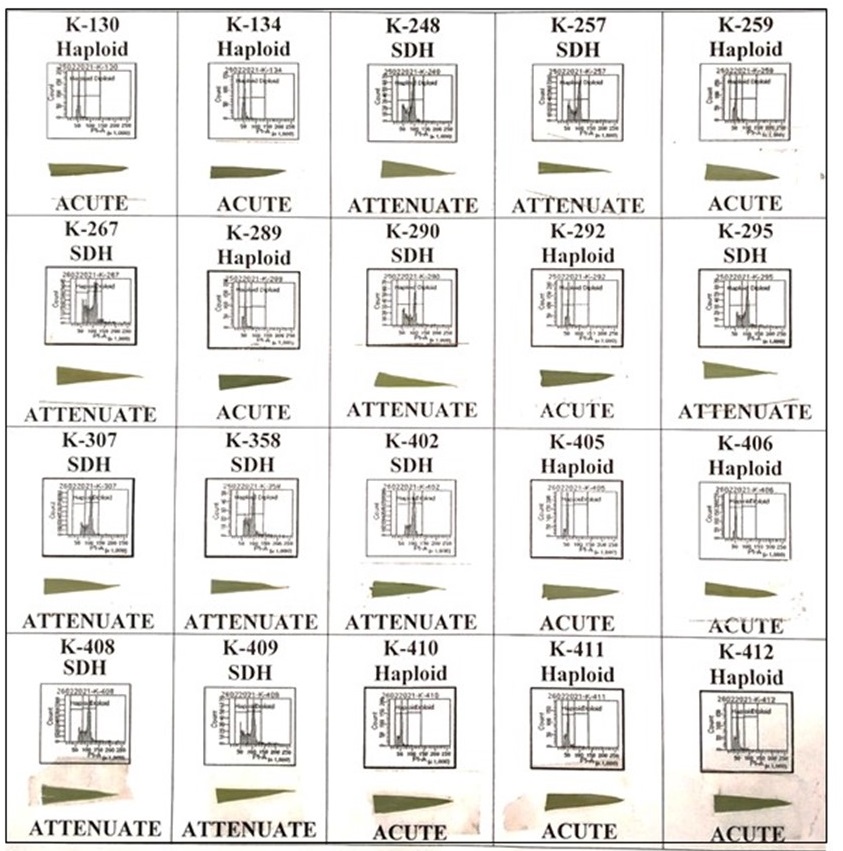

Supplement: Supplementary file 3 — Additional file 3: Fig. S3a. Representative samples of leaf apex shape of androgenically developed lines and their corresponding flow cytometry results. b Representative samples of leaf apex shape of androgenically developed lines and their corresponding ploidy level as determined by flow cytometry. [file 13007_2023_1085_MOESM3_ESM.jpg]

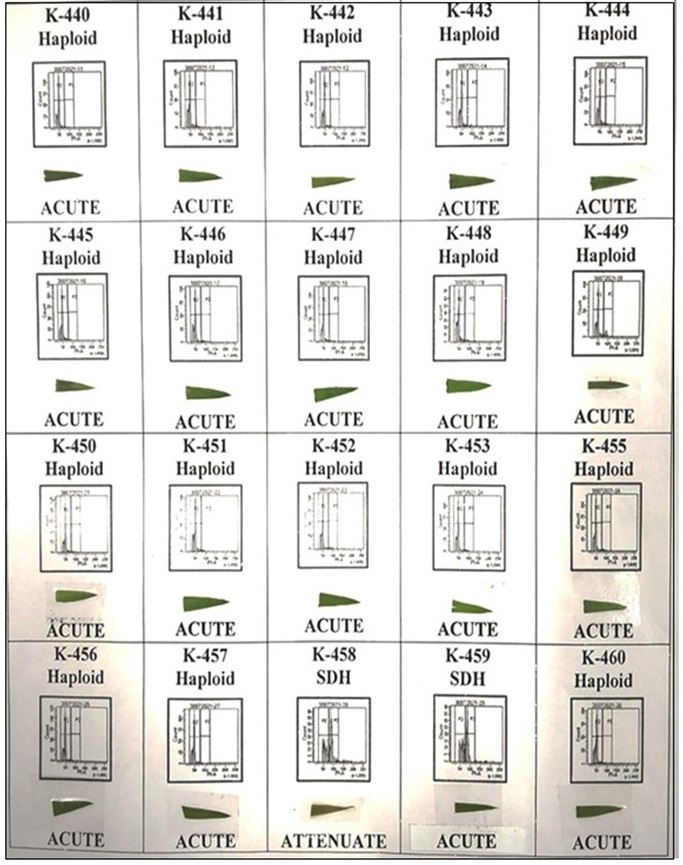

Supplement: Supplementary file 4 — Additional file 4: Fig. S4. Variations in physiological parameters a Photosynthetic rate b Stomatal conductance c Transpiration rate, on three consecutive days among the androgenically developed lines differing in leaf apex shape at early vegetative stage. [file 13007_2023_1085_MOESM4_ESM.jpg]

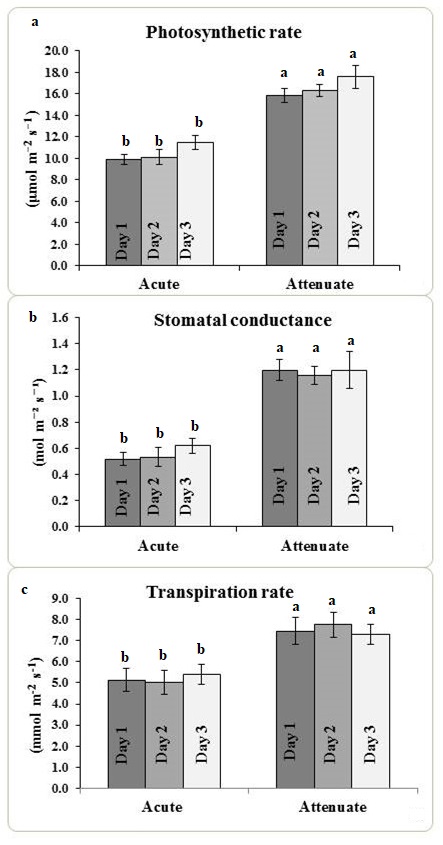

Supplement: Supplementary file 5 — Additional file 5: Fig. S5. Illustration of three important stages in rice doubled haploidy and significance of present study in increasing the efficiency of rice doubled haploids. [file 13007_2023_1085_MOESM5_ESM.jpg]

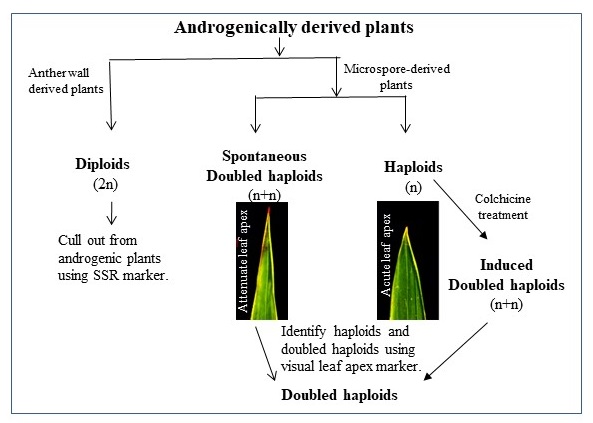

Supplement: Supplementary file 6 — Additional file 6: Table S1. Gas exchange parameters of androgenically developed lines differing in leaf apex shape at early vegetative stage. [file 13007_2023_1085_MOESM6_ESM.jpg]
