# Supplementary material for: A novel visual marker to distinguish haploids from doubled haploids in rice (Oryza sativa, L) at early growth stages
Source: Plant Methods. 2023 Dec 1;19:137. doi: 10.1186/s13007-023-01085-z (PMC10691067; doi:10.1186/s13007-023-01085-z)
Supplement: Supplementary file 7 — Additional file 7: Table S2. Comparison of visual (leaf apex) marker with two commonly used methods (flow cytometry and chromosome or plastid count) for identification of haploids in rice. [file 13007_2023_1085_MOESM7_ESM.docx]

**Table S2:** Comparison of visual (leaf apex) marker with two commonly used methods (flow cytometry and chromosome or plastid count) for identification of haploids in rice.

|  | **Visual marker**  **(Leaf apex shape)** | **Flow cytometry** | **Chromosome/plastid count** |
| --- | --- | --- | --- |
| Instrument | Not needed | Needs complex instrument | Needs microscope |
| Skill needed | Least skill | High level of skill | Moderate level of skill |
| Accuracy | 98 % | 100% | 100% |
| Expense | No investment needed | Expensive capital investment and expensive consumables | Expensive Microscope |
| Speed | Thousands of samples per day per person | Hundreds of samples per day per person | Hundreds of samples per day/person |
| Sample preparation, labeling of samples, and cross verification | Not needed. On-the-spot identification is possible | Needed | Needed |
